# Long range temporal correlations (LRTCs) in MEG-data during emerging psychosis: Relationship to symptoms, medication-status and clinical trajectory

**DOI:** 10.1016/j.nicl.2021.102722

**Published:** 2021-06-08

**Authors:** Gabriela Cruz, Tineke Grent-'t-Jong, Rajeev Krishnadas, J. Matias Palva, Satu Palva, Peter J. Uhlhaas

**Affiliations:** a Institute of Neuroscience and Psychology, University of Glasgow, Glasgow, United Kingdom; b Department of Child and Adolescent Psychiatry, Charité Universitätsmedizin, Berlin, Germany; c Neuroscience Centre, Helsinki Institute of Life Science, University of Helsinki, Finland; d Department of Neuroscience and Biomedical Engineering, Aalto University, Finland

**Keywords:** Schizophrenia, Emerging-psychosis, Magnetoencephalography, Oscillations, Longrange-temporal-correlations, Biomarker

## Abstract

Long-Range Temporal Correlations (LRTCs) index the capacity of the brain to optimally process information. Previous research has shown that patients with chronic schizophrenia present altered LRTCs at alpha and beta oscillations. However, it is currently unclear at which stage of schizophrenia aberrant LRTCs emerge. To address this question, we investigated LRTCs in resting-state magnetoencephalographic (MEG) recordings obtained from patients with affective disorders and substance abuse (clinically at low-risk of psychosis, CHR-N), patients at clinical high-risk of psychosis (CHR-P) (n = 115), as well as patients with a first episode (FEP) (n = 25). Matched healthy controls (n = 47) served as comparison group. LRTCs were obtained for frequencies from 4 to 40 Hz and correlated with clinical and neuropsychological data. In addition, we examined the relationship between LRTCs and transition to psychosis in CHR-P participants, and the relationship between LRTC and antipsychotic medication in FEP participants. Our results show that participants from the clinical groups have similar LRTCs to controls. In addition, LRTCs did not correlate with clinical and neurocognitive variables across participants nor did LRTCs predict transition to psychosis. Therefore, impaired LRTCs do not reflect a feature in the clinical trajectory of psychosis. Nevertheless, reduced LRTCs in the beta-band over posterior sensors of medicated FEP participants indicate that altered LRTCs may appear at the onset of the illness. Future studies are needed to elucidate the role of anti-psychotic medication in altered LRTCs.

## Highlights

• Long Range Temporal Correlations do not reflect the clinical trajectory of psychosis.
• Reduced Long Range Temporal Correlations may appear at the onset of psychosis.
• Antipsychotic medication may affect Long Range Temporal Correlations.
• Long Range Temporal Correlations do not correlate with symptoms of psychosis.

## 1 Introduction

Schizophrenia (ScZ) is a severe psychiatric condition that typically emerges during the transition from adolescence to adulthood (Uhlhaas and Singer, 2011) and is associated with a range of neurobiological and cognitive impairments (Insel, 2010). Until recently, pathophysiological theories have focussed on the crucial role of dopamine as a mechanism for the manifestation of psychotic symptoms, in particular hallucinations and delusions, and certain cognitive deficits associated with prefrontal cortex (Howes and Kapur, 2009). However, it is currently unclear whether aberrant dopaminergic neurotransmission is the primary disturbance since the cortex-wide occurrence of cognitive dysfunctions as well as basic circuit deficits are difficult to reconcile with the dopamine hypothesis (Kantrowitz and Javitt, 2010).

More recently, evidence has emerged that ScZ may fundamentally involve a disturbance in the balance between excitation and inhibition (E/I-Balance) (Grent-'t-Jong, Gross, et al., 2018). During normal brain functioning, efficient information transfer in neural networks is mediated by (GABA)ergic interneurons that regulate pyramidal cell activity, leading to rhythmic fluctuations in excitability (Kopell and LeMasson, 1994, Sohal et al., 2009). In ScZ, post-mortem (Lewis et al., 2012) as well as genetic data (Pocklington et al., 2015) have highlighted that rhythm-generating PV + interneurons and NMDA-Rs (Woo et al., 2004) are dysfunctional, leading to widespread disinhibition in neural circuits. The precise contributions of NMDA-Rs and GABAergic interneurons towards aberrant E/I-Balance in ScZ remains unclear, however. One possibility is that circuit deficits are due to a primary dysfunction in inhibitory interneurons in ScZ (Benes and Berretta, 2001). In addition, evidence exists that impaired inhibition could be the result of NMDA-R hypofunctioning on PV + interneurons (Woo et al., 2004) or reduced NMDA-R drive on pyramidal cells (Chung et al., 2016).

An important manifestation of aberrant E/I-balance are alterations in the temporal coordination of neuronal activity. In ScZ, there is consistent evidence that the amplitude as well as synchronization of neural oscillations at low and high-frequencies are impaired (Uhlhaas and Singer, 2010). Recent evidence suggests that impaired rhythmic activity is already present in participants who meet clinical high-risk criteria for psychosis (CHR-P) (Grent-‘t-Jong et al., 2020b, Grent-'t-Jong et al., 2018) as well as in patients with a first-episode of psychosis (FEP) (Grent-'t-Jong, Rivolta, et al., 2018).

An aspect of temporal processing of neural networks that has received less attention so far in ScZ are long-range temporal correlations (LRTCs) in neuronal activity. Fluctuations in neuronal oscillations in MEG/EEG data are governed by LRTCs, which persist from seconds to hundreds of seconds and which decay over time obey a power-law function (Lin et al., 2016, Linkenkaer-Hansen et al., 2001, Palva et al., 2013, Smit et al., 2013, Zhigalov et al., 2015, Zhigalov et al., 2017). Power-law scaling and LRTCs are suggestive of the neuronal networks operating in a critical state (Chialvo, 2010, Cocchi et al., 2017, Kello et al., 2010, Plenz and Thiagarajan, 2007, Shew and Plenz, 2013). Importantly, as predicted by the theoretical models, LTRCs index the efficiency of neural networks (Ma et al., 2019) and behavioral performance (Simola et al., 2017). Critical brain dynamics and LRTCs are controlled by the E/I-balance of the neuronal networks (Bruining et al., 2020, Li et al., 2020, Liang et al., 2020, Poil et al., 2012, Rubinov et al., 2011) of which alterations characterize several brain disorders (Bi et al., 2020, Bruining et al., 2020, Grent-'t-Jong et al., 2018, Uhlhaas and Singer, 2010).

Psychiatric disorders are indeed associated with altered autocorrelations in amplitude fluctuations. For instance, increased LTRCs characterize epilepsy (Monto et al., 2007) and Autism Spectrum Disorders (Bruining et al., 2020), while in Alzheimer’s Disease reduced LRTCs have been observed (Montez et al., 2009). In chronic ScZ patients, there is evidence that LRTCs at alpha-band (Alamian et al., 2020, Nikulin et al., 2012) and beta-band frequencies (Alamian et al., 2020, Moran et al., 2019, Nikulin et al., 2012, Sun et al., 2014) are attenuated. However, it is currently unclear whether aberrant LRTCs are already present during early illness stages and thus could constitute a potential biomarker for early detection of psychosis.

Early signs of psychosis as well as associated cognitive deficits are already present several years prior to the full emergence of schizophrenia (Fusar-Poli et al., 2013) and, therefore, research efforts have shifted the focus towards identifying circuit abnormalities and biomarkers in participants who are at-risk for the development of psychotic disorders. There is preliminary evidence that participants meeting clinical high-risk criteria for psychosis (CHR-P) are characterized by altered neural oscillations. In addition, patients with a first-episode of psychosis (FEP) are characterized by reductions in the amplitude and synchrony of high-frequency oscillations (Spencer et al., 2008).

To further characterize alterations of temporal processing in neural networks in emerging psychosis, we examined LRTCs in resting-state oscillations obtained from magnetoencephalographic (MEG) recordings in participants who met CHR-P (n = 115) and FEP-criteria (n = 25). Results were compared to matched healthy controls (CTRL) (n = 47). In addition, a group of patients with affective disorders and substance abuse (non-psychotic disorders, CHR-N) (n = 38) was also compared against the CTRL group. Based on previous evidence of impaired oscillatory activity in CHR-P and FEP populations, we predicted attenuated LRTCs in alpha and beta frequency bands, while participants with affective disorders and substance abuse would be intact.

## 2 Materials and methods

### 2.1 Participants

A total of 236 MEG-data sets from participants were analysed. 11 participants were excluded during pre-processing, the remaining 225 data-sets were divided into four groups: (1) n = 115 participants meeting CHR-P criteria, (2) 38 participants that did not meet CHR-P criteria (CHR-N) but were characterized by non-psychotic disorders, such as affective disorders and substance abuse (3) 25 patients with FEP (12 antipsychotic-naïve) and, (4) 47 healthy control participants (CTRL) without an axis I diagnosis or family history of psychotic disorders. CHR-P and CHR-N participants were recruited from the Youth Mental Health Risk and Resilience (YouR) Study (Uhlhaas et al., 2017).

Participants in the CHR-P group met ultra-high risk criteria according to the Comprehensive Assessment of At-Risk Mental States (CAARMS) Interview (Yung et al., 2005) and the Cognitive Disturbances (COGDIS) and Cognitive-Perceptive (COPER) basic symptoms criteria according to the Schizophrenia Proneness Instrument, Adult version (SPI-A) (Schultze-Lutter et al., 2007). FEP-patients were assessed with the Structured Clinical Interview for DSM-IV (SCID) (see Table 1) (First and Spitzer, 1995) and with the Positive and Negative Symptom Scale (PANSS) (Kayet al., 1987). For all groups except FEP-patients, cognition was assessed with the Brief Assessment of Cognition in Schizophrenia (BACS) (Keefe et al., 2004) (see Table 1).

**Table 1:** Demographic and Clinical data.

	CTRL	CHR-N	CHR-P	FEP	Group effect* ^o^	Pairwise comparisons
Age (mean/SEM)	22.8/3.7	22.5/4.6	21.9/4.5	23.8/4.1	H(3) = 8.24 P = 0.04	CHR-P vs. FEP, p = 0.05
Male/Female	17/31	27/10	32/82	16/9	X^2^ (3) = 12.3, P = 0.006	CTRLS vs. FEP, P = 0.019 CHR-N vs. FEP, p = 0.003 CHR-P vs. FEP, p = 0.0007
Education (mean/SEM)	16.8/3.2	16.2/3.2	15.1/3.2	14.4/3	H(3) = 9.82 P = 0.2	CTRL vs. CHR-P, p = 0.01
BACS (mean/SEM)						
Verbal Memory	−0.03/1.01	0.04/1.13	−0.33/1.26	–	n.s.	–
Digit Sequencing	−0.08/0.92	0.15/1.21	−0.11/1.43	–	n.s.	–
Motor Speed	−0.06/1.05	−0.61/1.18	−1.04/1.31	–	H(2) = 19.36 P < 0.0001	CTRL vs. CHR-P, p < 0.0001
Verbal Fluency	0.01/0.99	−0.20/1.00	−0.09/1.22	–	n.s.	–
Symbol Coding	−0.04/0.94	−0.00/1.32	−0.60/1.14	–	H(2) = 15.27 P = 0.0005	CTRL vs. CHR-P, p = 0.002 CHR-P vs. CHR-N, p = 0.01
Executive Function	−0.02/0.99	0.16/1.29	−0.19/1.39	–	n.s.	
Composite Score	−0.07/0.98	−0.11/1.18	−0.64/1.36	–	H(2) = 8.75 P = 0.01	CTRL vs. CHR-P, p = 0.03
CAARMS	0.7/2.4	6.4/6.1	29.1/17.9	–	H(2) = 113.36 P < 0.00001	CTRL vs. CHR-N, p < 0.0001 CTRL vs. CHR-P, p = 0.03 CHR-P vs. CHR-N, p < 0.0001
PANSS						
Positive	–	–	–	17.8/7.3	–	–
Negative	–	–	–	15.2/9.4	–	–
PANSS_C	–	–	–	19.9/9.3	–	–
Excitement	–	–	–	8.7/4.3	–	–
Disorganized	–	–	–	11.7/5.9	–	–
Total	–	–	–	73.4/28.9	–	–
Medication						
None	46	26	57	3	–	–
Antidepressants	1	10	47	3	–	–
Antipsychotics	0	0	3	13	–	–
Mood-stabilizer	0	0	4	0	–	–
Anxiolytics	0	0	1	1	–	–
Other(unknown)	0	0	5	0	–	–

*Kruskal-Wallis independent-sample test, Alpha-level 0.05. ^o^Chi-Squared test for gender comparisons.

The study was approved by the ethical committees of University of Glasgow and the NHS Research Ethical Committee Glasgow & Greater Clyde. All participants provided written informed consent, including consent to use anonymised data in future research.

### 2.2 Follow-Up data

Participants meeting CHR-P criteria were re-assessed at 3, 6, 9, 12, 18, 24, 30, and 36 months intervals to examine transition to psychosis. Criteria for transition to psychosis were defined on the basis of the CAARMS symptom scores of sufficient duration and frequency, using symptom severity scores of 6 (maximum) on unusual thought content, non-bizarre ideas, or disorganized speech, or a score of 5–6 on perceptual abnormalities. Associated frequency scores should be ranging 4–6, with experiences lasting longer than one week. When transition to psychosis was thus confirmed, a SCID Interview was conducted to establish the DSM-IV-category of the psychotic disorder.

### 2.3 MEG recording

All participants undertook a 5-minute eyes-open resting-state baseline MEG recording at the Centre for Cognitive Neuroscience and Imaging (CCNi), University of Glasgow. Data was acquired using a 248-channel 4D-BTI magnetometer system (MAGNES 3600 WH, 4D-Neuroimaging, San Diego), recording at a sampling frequency of 1017.25 Hz, low-pass filtered at 400 Hz. During the recording, participant were asked to focus on a fixation cross and to not think of anything in particular (“blank state of mind”).

### 2.4 MEG data analysis

**Fig. 1:**
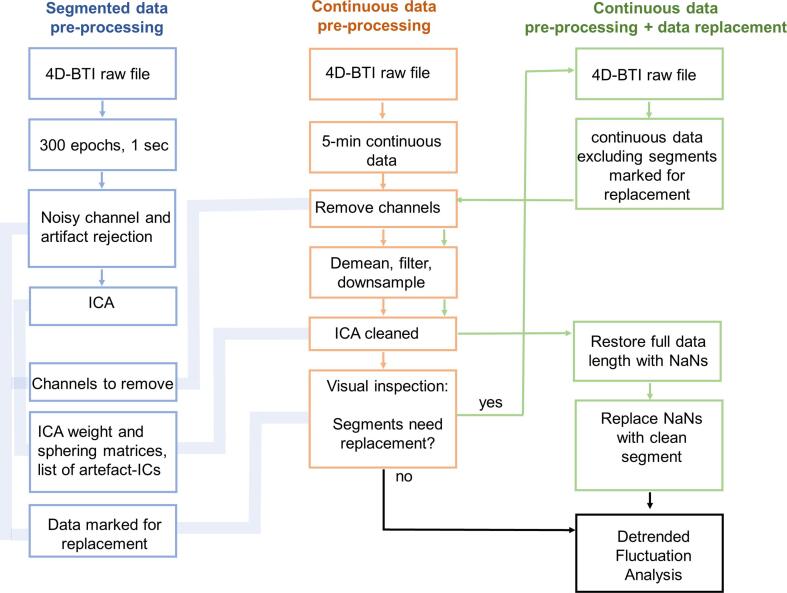
Pipeline for data pre-processing. Data was first pre-processed (left column) to obtain ICA weight and sphering matrices plus a list of noisy channels and data points to replace. Continuous data (middle column) was cleaned using the information previously obtained from segmented data. In order to preserve the length of the original data, some minor artifacts were kept and high-amplitude artifacts plus high-frequency noise that were not removed with low-pass filtering, were replaced (right column).

MEG data pre-processing and analyses were performed using Fieldtrip and custom MATLAB scripts. Pre-processing aimed at maintaining the original length of the time series, as full-length continuous data was subjected to Detrended Fluctuation Analysis (DFA) to estimate LRTCs. On an initial step, segmented data was pre-processed to obtain the following information: sample points containing artifacts, Independent Component Analysis (ICA) matrices, a list of artifact-ICs and a list of artifactual channels for rejection. This information was saved and later used to clean the continuous data, as well as to perform data replacement for those time series contaminated with high-amplitude noise (Fig. 1). Specific pre-processing steps are described below.

4D-BTI data files were loaded into MATLAB and 300 epochs of 1-second duration were created (offline: demeaned, 49–51 Hz third-orde Butterworth notch filter, down-sampled to 400 Hz). Noisy channels and epochs containing muscle or high-amplitude artifacts were rejected with a semi-automatic approach, that is, bad segments were first flagged with the Fieldtrip function FT_REJECTARTIFACT and then visually inspected. A final outlier detection was performed using the function FT_REJECTVISUAL. During visual inspection, segments containing high amplitude-noise were marked for replacement and their sample information was stored to be used during continuous data pre-processing. The segmented clean time series were high-pass filtered using the fieldtrip function FT_PREPROCESSING (1 Hz, 3rd order, Butterworth filter) and submitted to Independent Component Analysis (ICA). The resulting weight and sphering matrices, along with a manual registration of artifact-ICs, were saved to later remove eye-movement and heartbeats from continuous data (a median of 3 ICs were removed per dataset, most of the datasets had<7 ICs removed).

In the next step, 5-minute continuous data were loaded into MATLAB, noisy channels previously identified were automatically removed and the data was then demeaned. To eliminate 50 Hz line noise a stopband IIR filter was designed with the butter() function in MATLAB, and applied to the data using the filtfilt() function (49–51 Hz, third-order Butterworth notch filter). Then a Hamming high-pass FIR filter was designed with the MATLAB function fir1() (cut-off: 1 Hz, filter order: 3052, sampling frequency: 1017.25 Hz) and applied to the data using a custom FFT filter. The data was then down-sampled to 400 Hz. ICA mixing matrices were loaded and applied to the continuous data; previously identified artifact-ICs were subsequently removed. The data was then lowpass filtered for visualisation purposes only (40 Hz, 3rd order, Butterworth low-pass filter) and visually inspected. Segments marked for rejection during the ICA-cleaning process were highlighted to facilitate identification of segments to replace. If data did not require replacement, it was saved for DFA. If data replacement was required, the following steps were followed: the sample information of the artifacts was used to read-in the raw data excluding the artifactual parts – this step is relevant to avoid additional long-lasting artifacts, produced by applying pre-processing steps on continuous data containing high amplitude noise, such as SQUID jumps. Then, all pre-processing steps already described were applied. To do data replacement, the original length of the data was reconstructed filling the artifactual sample points with NaNs. A Savitzky-Golay finite impulse response (FIR) smoothing filter (filter order:1, frame length: 41) was applied to the data containing NaNs to obtain the trend of the signal (steady-state portion of the filtered signal), and the missing segment was completed with the interp1() MATLAB function, using the shape-preserving piecewise cubic interpolation method. The interpolated values were used to re-trend and insert a clean portion of the data, avoiding edge artifacts (see suppl. Fig. 1). This process replaced SQUID-jumps and some muscle artifact (visible after lowpass filtering) with clean data ready for DFA. The median of the total length of data replaced by subject was 0.8 s (suppl. Fig. 2). The median of the longest segments replaced was 0.5 s, only 4 subjects had more than 4 continuous seconds replaced, there were no differences between groups (Kruskal-Wallis independent-sample test, H = 6.16, P = 0.1) (suppl. Fig. 3). Eleven out 236 participants were excluded from the study: five datasets still presented heartbeats after ICA cleaning, two required high number of ICs removed and data was still noisy (15 and 16 ICs removed, median was 3), one presented several dead channels after ICs removal and three were excluded because of high frequency noise that could not be filter out, several artifacts across the data and large head movements. In total 225 datasets were subjected to DFA analysis.

**Fig. 2:**
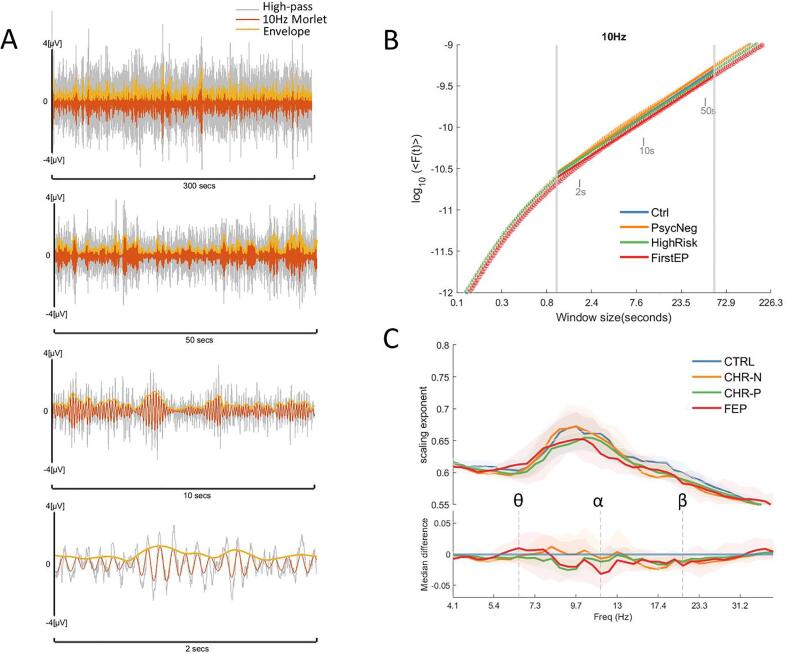
Power-law decaying narrow-band oscillations during resting state. (A) In this example, the artifact-free-high-pass filtered signal (grey) was convoluted with a complex 10 Hz Morlet wavelet (orange). The absolute values of the analytic signal (i.e., envelope of the oscillation, yellow line) were analysed with Fourier Detrended Fluctuations Analysis (F-DFA). This filtering process was repeated for all frequencies in the Bank of Morlet wavelets (4 – 40 Hz in log10scale). The aim of F-DFA is to evaluate the presence of long-range temporal (auto) correlations (LRTCs) and the speed of their decay over time. We calculated a set of 181 window sizes equally spaced on a log10 scale between 0.08 and 300 secs, examples of windows of size 2, 10 and 50 s are shown in the figure. F-DFA method calculated fluctuations on the frequency domain for each window size and the resulting F(t) were plotted against its correspondent window size on a log–log scale (B). The scaling exponents correspond to the slope α of the power-law function F(t) and represent how strongly correlated is the signal. The lower the scaling exponent (slope closer to 0.5), the faster the autocorrelation decay, meaning that the signal is governed by uncorrelated random processes. To avoid strong autocorrelations induced by the filter, the slope was calculated using window sizes between 1 and 60 s (fitting range indicated with vertical grey lines). (C) The resulting scaling exponents median collapsed across all channels were plotted for each group across all frequencies (4 to 40 Hz), shaded areas represent bootstrapped (n = 5000) 95% confidence intervals. The bottom panel depicts the median difference for each clinical group against the controls. The greatest difference between controls and any clinical group revealed three frequencies of interest, θ = 6.5 Hz, α = 11.6 Hz and β = 20.7 Hz, which were subjected to a non-parametric permutation (n = 5000) *t*-test (in Fig. 3). (For interpretation of the references to colour in this figure legend, the reader is referred to the web version of this article.)

**Fig. 3:**
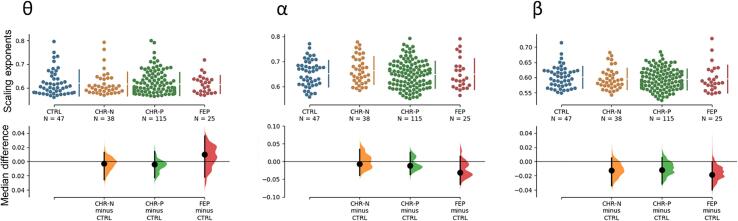
Scaling exponents collapsed across channels for each group at the frequencies of interest. Scaling exponents at theta, alpha and beta were subjected to a non-parametric permutation (n = 5000) *t*-test. Dots in the upper part of the plots represent single subject scaling exponent values (collapsed across channels), revealing the sample distribution. The bottom panels show the size of the difference between clinical groups and controls. The 95% confidence interval is represented with the black vertical lines. Shades to the right of each confidence interval represent the distribution of the resampled median differences. 95% confidence intervals of the resampling distribution were built via bias-corrected and accelerated bootstrap correction, to account for skew data distribution.

### 2.5 Detrended fluctuation analysis (DFA)

DFA evaluates LRTCs (i.e. autocorrelation properties of a time series) in spontaneous brain oscillations at different time scales, offering an index of how autocorrelations decay over time. If the data present LRTCs, the result of the DFA is a value α between 0.5 and 1, indicating that the time series are autocorrelated, such that large fluctuations are likely to be followed by large fluctuations and small fluctuations are likely to be followed by small fluctuations. If α is equal to 0.5, indicates that the time series are uncorrelated, thus the closer α get to 0.5 (lower LRTCs), the faster is the autocorrelation decay, indicating that time series poses greater random variability.

The first step to calculate α was to filter the high-pass-artefact-free time series into 40 frequencies using a bank of Morlet wavelets equally spaced on a log10 scale between 4 and 40 Hz. Data above 40 Hz was not considered in the analysis because of noisy power spectrums in a high proportion of participants that did not improve after the cleaning process. Next, a set of T window sizes (n = 181) were defined on a log10 scale, ranging from 0.08 to 300sec. The absolute value of the analytic signal (i.e. the envelope of the signal) was extracted for each time series and submitted to DFA (for an example of window sizes and their envelope see Fig. 2A). This process resulted on a series of fluctuation functions F(t) for each window size t ∈ T for each Morlet frequency. The Fourier-DFA method applied here calculates fluctuations on the frequency domain using a Gaussian kernel for detrending (Nolte at al., 2019), unlike the classical DFA approach that calculates fluctuations on the amplitude of the envelope using a linear detrending. F(t) was plotted on a log–log scale for each window size. DFA scaling exponents were estimated as the slope α of the power law function F(t) via bisquare robust fit linear regression. The fitting range included window sizes between 1 and 60 s: data filtering induce strong autocorrelations (Hardstone et al., 2012), as it can be directly observed in Fig. 2B. Filter-induced correlations are revealed through a stronger slope in the power law function below 1 s (suppl. Fig. 4). Therefore, to avoid the influence of filtering the lowest boundary of the fitting range was set to 1-second. The upper limit of the fitting range was 60 s, corresponding to 20% of the available data.

**Fig. 4:**
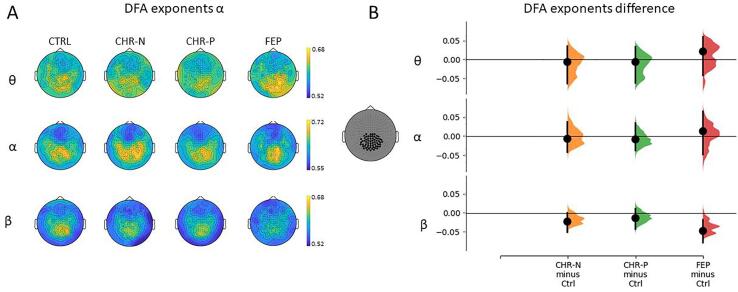
(A) Topographic distribution of DFA exponents for each group and frequency of interest. (B) The non-parametric permutation (n = 5000) *t*-test was applied to a posterior selection of channels. The 95% confidence interval of the difference between clinical groups and controls is represented with the black vertical lines. The distribution of the bootstrapped (n = 5000) median difference between the FEP group and controls at the beta band β, is significatively below the control group median (p = 0.0142, uncorrected).

### 2.6 Statistical analysis

To identify at which frequency scaling exponents CHR-N, CHR-P and FEP-groups deviated from controls, the difference of the median across all frequencies were obtained (Fig. 2C). Next, the frequency depicting the largest effect size for any group were identified within theta (4–8 Hz), alpha (8–12 Hz) and beta (12–40 Hz) bands. This approach yielded three frequencies of interest at 6.5 Hz, 11.6 Hz and 20.7 Hz. Single subject DFA values (median across all channels) was plotted to reveal the underlying distribution of each group. To evaluate the magnitude of the effect size and its precision each participant’s median difference (at each frequency of interest) were subjected to a non-parametric permutation (n = 5000) *t*-test against the null hypothesis of no difference with the CTRL group. Confidence intervals (CI) were obtained by selecting the central 95% of the resampling distribution. Bias-corrected and accelerated bootstrap correction was applied to account for possible skew data distribution (Ho et al., 2019). The P values reported represent the likelihood of observing the effect size, if the null hypothesis of zero difference is true. The effect sizes and CI are reported as: effect size [CI width lower bound, upper bound]. We followed this approach because it allows to clearly quantify the effect size of the difference (Ho et al., 2019). Previous publications comparing scaling exponents of schizophrenic patients with controls, revealed relatively small effect size of the difference (around 0.05) (Moran et al., 2019, Nikulin et al., 2012). Thus, with this approach we can reliably estimate the effect size and their certainty, in order to understand how much the clinical groups differ from controls.

A pairwise linear correlation between median scaling exponents and power of the signal was also carried out. It is expected that no correlation between power of the signal and its corresponding DFA exponents would be observed (Linkenkaer-Hansen et al., 2001, Linkenkaer-Hansen et al., 2007). Nevertheless, correlations can arise when extrinsic noise (uncorrelated by definition) is high relative to the neuronal signal power, the resulting signal is artifactually more uncorrelated, leading to DFA exponents closer to 0.5. In our data, the correlation between signal power and scaling exponents was low but significant when all channels were considered. Therefore, as a next step, scaling exponents were plotted over a topographic representation and a selection of posterior channels - whose correlation with power was no longer significant – was performed. The same non-parametric permutation (n = 5000) *t*-test described above was applied to investigate the median difference in the subset of posterior channels. Statistical tests were performed in Python using the code available from https://github.com/ACCLAB/DABEST-python.

## 3 Results

### 3.1 Demographics and clinical data

There were no age differences between CHR-N, CHR-P and FEP participants and the control group (Table 1). Only FEP participants were older compared to CHR-P. There were significantly more females relative to the FEP group in the CHR-P, CHR-N and CTRL groups, and CHR-P participants had less years of education relative to controls. In terms of clinical scores, CHR-P patients were characterized by lower performance on the Motor Speed, Symbol Coding subscales and composite BACS scores relative to the CTRL group.

### 3.2 LRTCs are not a feature of the clinical trajectory of psychosis

Participants from the FEP groups depicted the greatest median differences in scaling exponents relative to controls across the three frequencies of interest. However, none of these differences were significant (Fig. 3). Theta: Δmedian = 0.009 [95.0%CI −0.022, 0.036], p = 0.52, Alpha: Δmedian = -0.031 [95.0%CI −0.064, 0.014], p = 0.36 and Beta: Δmedian = -0.018 [95.0%CI −0.039,-0.001], p = 0.11. Likewise, scaling exponents for the CHR-P group did not differ from controls at, theta (Δmedian = -0.004 [95.0%CI −0.002, 0.014], p = 0.46), alpha (Δmedian = -0.012 [95.0%CI −0.36, 0.026], p = 0.47) nor at beta (Δmedian = -0.011 [95.0%CI −0.031, 0.006], p = 0.24). The CHR-N group did not differ from the CTRL group either; theta (Δmedian = 0.002 [95.0%CI −0.025, 0.012], p = 0.81), alpha (Δmedian = 0.007 [95.0%CI −0.038, 0.034], p = 0.79) and beta (Δmedian = −0.013 [95.0%CI −0.034, 0.005], p = 0.18).

### 3.3 Reduced LRTCs may appear at the onset of the psychosis

The comparison averaged across all channels between clinical groups and controls did not reveal a significant difference. However, as the whole-brain analyses can dismiss a possible local effect we decided to investigate a smaller subset of posterior channels. Scaling exponents for the FEP group decreased significantly from controls at the beta frequency (Δmedian = -0.046 [95.0%CI −0.08,-0.02], p = 0.016, uncorrected), while no difference from controls was observed at alpha (Δmedian = -0.014 [95.0%CI −0.046, 0.065], p = 0.60, uncorrected) nor at theta (Δmedian = 0.022 [95.0%CI −0.041, 0.059], p = 0.68, uncorrected) (Fig. 4). Nor significant effects were found for any of the other groups.

### 3.4 Relationship between LRTCs and spectral power

**Fig. 5:**
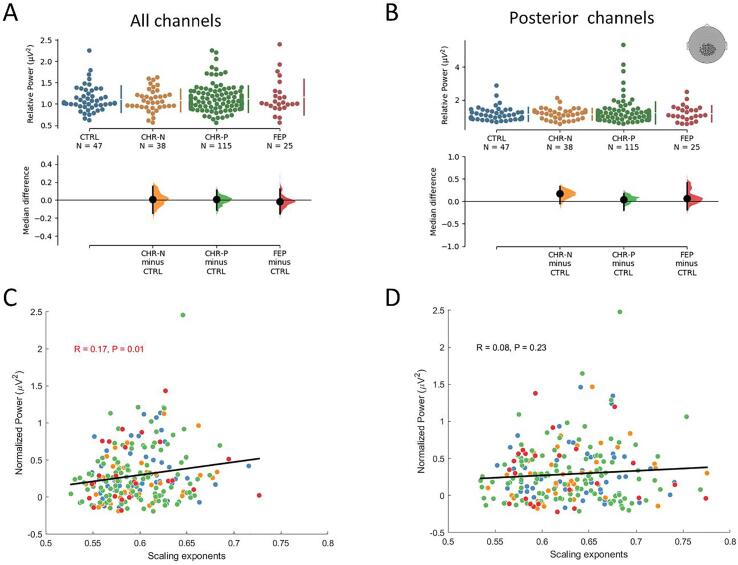
Beta relative power of the clinical groups do not differ from controls when averaged across all channels (A) or only across a posterior channel selection (B). The correlation between signal power and scaling exponents for all channels was significant but marginal (C), whereas no correlation was observed for the posterior channel selection (D). Power spectrum was normalised, given that absolute power levels for some participants were greater in several orders of magnitude.

To draw conclusions in relation to differences in scaling exponents between groups, it is necessary to confirm that the scaling exponent are not related to changes in oscillatory power. The relative power of beta (Fig. 5A), theta or alpha (suppl. Fig. 5) oscillations, however, did not differ across groups. Furthermore, the correlation between signal power and scaling exponents was marginal but significant when all channels were considered, likely because frontal channels usually present lower SNR in M/EEG recordings. The correlation was no longer significant when only the posterior channels selection was considered (Fig. 5B), accounting for only 8% of the scaling exponents variance across all groups.

### 3.5 Effects of antipsychotic medication on LRTCs in FEP-Group

**Fig. 6:**
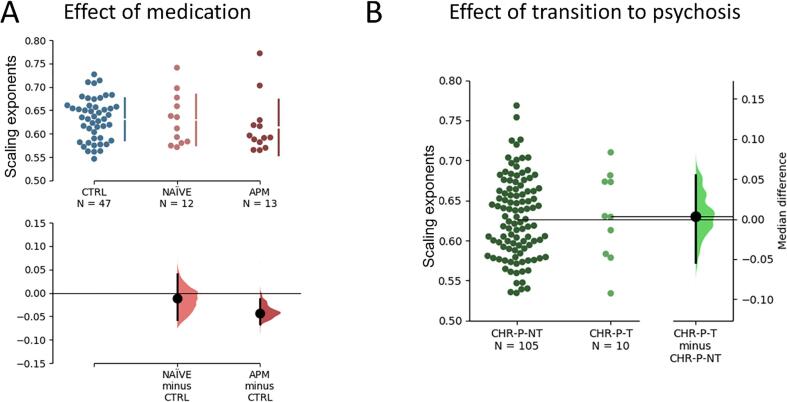
(A) The comparison between FEP-medication-naïve (NAIVE) and FEP-with-antipsychotic-medication (APM) did not reveal significant differences. However, only FEP-patients under APM were characterized by attenuated LRTCs compared to controls. (B) DFA Scores in Transitioned vs. Non-Transitioned CHR-Ps. The median difference between transitioned (CHR-P-T) and non-transitioned (CHR-P-NT) participants was close to zero (n.s.). Shades to the right of each pairwise comparison represent the distribution of the resampled median diferences.

We examined if antipsychotic medication (APM) status is associated with attenuated LRTCs observed at 20.7 Hz. Our results do not show a significant difference between medication-naïve and medicated FEP-patients (Δmedian = -0.031 [95.0%CI −0.086, 0.014], p = 0.19). However, FEP-patients with APM showed overall lower DFA scores (Δmedian = -0.04 [95.0%CI −0.066, −0.013], p = 0.023) (Fig. 6A).

### 3.6 Follow-Up data

We further evaluated whether participants in the CHR-P group who transitioned to psychosis (CHR-P-T: n = 10) showed decreased LRTCs at 20.7. The results showed that DFA scores did not differ between the two groups (Fig. 6B).

### 3.7 Correlation between DFA scaling exponents and clinical scores

We investigated whether psychopathological and neurocognitive measures correlated with the DFA scaling exponents at the three frequencies of interest (6.5 Hz, 11.6 Hz and 20.7 Hz). There were no significant correlations between neurocognitive scores (BACS-composite score, BACS individual test scores) and DFA-scaling exponents (Table 2). In addition, no significant correlations were observed between DFA-scaling exponents and clinical symptoms in the CHR-P (CAARMS-total scores, CAARS-Subscales) and in the FEP-group (Total PANSS, Positive, Negative, Excitement, Disorganisation Scores). Only verbal memory correlated negatively in the CTRL group which was, however, not significant following correction for multiple comparisons.

**Table 2:** Spearman correlation values for psychopathological and neurocognitive measures and DFA scaling exponents at the three frequencies of interest by group.

		CTRL			CHR-N			CHR-P			FEP	
	6.5 Hz	11.6 Hz	20.7 Hz	6.5 Hz	11.6 Hz	20.7 Hz	6.5 Hz	11.6 Hz	20.7 Hz	6.5 Hz	11.6 Hz	20.7 Hz
BACS												
Verbal Memory	−0.29	−0.19	−0.07	−0.01	−0.30	0.03	0.02	0.06	−0.04	–	–	–
Motor Speed	−0.21	−0.23	−0.05	−0.00	0.19	0.16	0.06	−0.07	−0.10	–	–	–
Verbal Fluency	−0.12	−0.17	−0.09	0.27	−0.12	0.12	0.06	0.14	0.13	–	–	–
Symbol Coding	−0.07	−0.00	0.15	−0.14	−0.14	0.08	0.08	0.10	0.08	–	–	–
Executive Function	−0.03	0.14	−0.07	0.28	−0.12	0.20	0.11	−0.05	−0.15	–	–	–
Total Score	−0.30*	−0.14	−0.05	0.07	−0.18	0.21	0.11	0.07	−0.04	–	–	–
CAARMS	–	–	–	0.14	0.00	0.01	0.14	0.00	0.01	–	–	–
PANSS										0.07	0.04	0.02
Positive	–	–	–	–	–	–	–	–	–	0.02	0.27	0.24
Negative	–	–	–	–	–	–	–	–	–	0.12	−0.02	0.01
PANSS_C	–	–	–	–	–	–	–	–	–	−0.03	−0.05	0.02
Excitement	–	–	–	–	–	–	–	–	–	−0.04	0.06	0.01
Disorganized	–	–	–	–	–	–	–	–	–	0.06	0.05	0.07
Total	–	–	–	–	–	–	–	–	–			

*p = 0.039, uncorrected. All correlations were performed with normalized BACS-scores.

## 4 Discussion

The current study examined alterations in LRTCs in MEG-data from CHR-P participants and FEP-patients to establish whether changes in the 4–40 Hz frequency band may already be present during emerging psychosis. Our findings show that LRTCs in the clinical groups are similar with controls. In addition, preliminary analysis showed that LRTCs did not predict transition to psychosis in participants at higher risk of developing psychosis (CHR-P). LRTCs were neither correlated with clinical symptoms. Thus, impaired LRTCs are not a feature that reflects the clinical trajectory towards psychosis. Furthermore, our results support the idea that reduced LRTCs are rather a feature that can appear at the onset of psychosis, as the FEP-group showed a specific alteration in beta-band LRTCs over posterior regions. These reduced LRTCs appear to be driven by participants under anti-psychotic medication. Accordingly, these findings highlight that alterations in LRTCs represent a marker of aberrant temporal organisation of manifest psychosis and not a potential biomarker for early detection and diagnosis in CHR-P participants.

Recent evidence has suggested that emerging psychosis is associated with increased excitatory drive possibly the resulting of N-methyl-D-aspartate receptor (NMDA-R) hypofunctioning which leads to disinhibition in neural circuits (Krystal et al., 2017, Lisman et al., 2008). This is supported by recent findings with fMRI indicating that connectivity patterns in resting-state fMRI-data during the early phases of schizophrenia differ significantly from those observed in chronic patients (Anticevic et al., 2012, Anticevic et al., 2015a). Importantly, connectivity alterations during early illness stage closely corresponded to changes observed following the administration of the NMDA-R antagonist Ketamine in healthy volunteers (Anticevic, Corlett, et al., 2015). In addition, evidence from our group (Grent-'t-Jong, Gross, et al., 2018) has indicated that CHR-P participants and FEP-patients are characterized by increased gamma-band activity across cortical regions. The upregulation of gamma-band power in at-risk participants furthermore correlated with increased glutamate levels while GABA-levels were in the normal range, highlighting a possible shift towards increased excitatory drive during early illness stages.

A main finding from our data is that participants meeting CHR-P criteria were not characterized by alterations in LRTCs. Identification of potential biomarkers for early diagnosis and detection of psychotic disorders is an important objective of current research (Mikanmaa et al., 2019). Indeed, studies with EEG and MEG have identified abnormalities in neural oscillations in both resting-state as well as task-related contexts at low and high-frequencies in CHR-P participants (Grent-'t-Jong et al., 2018, Hamm et al., 2011, Hirvonen et al., 2017, Kwon et al., 1999). In addition, previous findings from our group (Grent-'t-Jong et al., 2020b) have shown that altered neural oscillations and their synchronization predicted persistence of attenuated psychotic symptoms (APS) and conversions to psychosis in CHR-P participants (Grent-'t-Jong et al., 2020a). The current study demonstrated, however, that attenuated LRTCs are not a present in groups at risk of developing psychosis.

Despite not observing significant alterations in LRTCs, we cannot rule out the possibility of reduced LRTCs for the FEP group relative to controls. A subset of posterior channels in the FEP group showed attenuated LRTCs at 20 Hz. The p-value of this difference is above correction for multiple comparisons. However, our analysis showed that the effect size of the difference (0.046) is close to differences reported in previous analyses of LRTCs in chronic schizophrenia. Specifically, two studies have reported decreased LRTCs at beta band at sensor level using electroencephalographic recordings (Moran et al., 2019, Nikulin et al., 2012) with effect sizes between 0.05 and 0.06 respectively. Reduced LRTCs at the beta band in patients with schizophrenia have also being confirmed using magnetoencephalographic recordings (Alamian et al., 2020), suggesting that attenuated LRTCs in the beta band is a robust feature of altered network activity in established schizophrenia. Nevertheless, reduced LRTCs seem to appear already at the onset of psychosis. Future research needs to confirm this finding and elucidate whether alterations in LRTCs follow disease evolution, either driven by intrinsic progressive pathology or by medication effects. Our data seem to indicate that medication may be playing a role, since medication-naïve FEP-patients were characterized by less pronounced deficits beta-band LRTCs, although previous research has indicated that reduced LRTCs are not associated with anti-psychotic medication. Further studies need to address this question, ideally in longitudinal design that involves the measurement of LRTCs prior initiation of antipsychotic medications in FEP-patients.

The current findings may also have implications for the current models of circuit dysfunctions in schizophrenia, specifically those implicating a shift in E/I-balance during emerging psychosis (Krystal et al., 2017). Autism Spectrum Disorders and Epilepsy, two disorders that involve excessive excitation, are characterized by increased LRTCs (Bruining et al., 2020, Monto et al., 2007) but see for a different finding (Jia and Yu, 2019). Accordingly, the current data indicate a possible failure in FEP-patients to sustain temporal patterning which could be due to the presence of elevated noise (Saunders et al., 2012) or a failure of inhibition (Lewis, 2014). Moreover, this pattern is consistent with a large body of work which has demonstrated impairments in synchronization in local and large-scale networks in schizophrenia (Spencer et al., 2003, Uhlhaas and Singer, 2010). Moreover, we examined the contribution of differences in spectral power towards alterations in LRTCs and showed that DFA scaling exponents did not correlate with power, indicating that decreased LRTCs in the FEP group cannot be explained by changes in the power spectrum of the signal. Furthermore, DFA scaling exponents did not correlate with clinical scores, replicating previous findings (Alamian et al., 2020, Moran et al., 2019).

## 5 Limitations

The rate of transition to psychosis in our CHR-P sample is currently lower than in previous studies (Fusar-Poli et al., 2013). Accordingly, the question whether LRTCs are predictive for transition to psychosis needs to be replicated in a larger sample of CHR-Ps. In addition, we did not assess frequencies above 40 Hz for the presence of LRTCs.

## 6 Conclusion

We provide novel data that altered LRTCs are not a biomarker that predicts transition to psychosis. These results contrast with our initial hypothesis, as we found that LRTCs across clinical groups were similar to controls. However, a local effect in the beta-band of FEP-patients indicates that reduced LRTCs may appear at the onset of psychosis, extending previous evidence for impaired LRTCs in chronic schizophrenia. Further studies need to confirm this finding and clarify the relationship between antipsychotic medication and attenuated LRTCs. These data highlight the need to develop more sensitive non-invasive measures to examine changes in E/I-balance for the characterization of circuit dysfunctions in psychosis and related disorder that could potentially inform the development of biomarkers and insights into pathophysiological mechanisms.

## CRediT authorship contribution statement

**Gabriela Cruz:** Conceptualization, Methodology, Software, Formal analysis, Writing - original draft, Visualization. **Tineke Grent-’t-Jong:** Investigation, Data curation. **Rajeev Krishnadas:** Investigation. **Matias Palva:** Funding acquisition, Conceptualization, Methodology, Writing - original draft. **Satu Palva:** Funding acquisition, Conceptualization, Methodology, Project administration, Writing - original draft. **Peter J. Uhlhaas:** Funding acquisition, Writing - review & editing.

## Declaration of Competing Interest

The authors declare that they have no known competing financial interests or personal relationships that could have appeared to influence the work reported in this paper.
