# Supplementary material for: Long range temporal correlations (LRTCs) in MEG-data during emerging psychosis: Relationship to symptoms, medication-status and clinical trajectory
Source: Neuroimage Clin. 2021 Jun 8;31:102722. doi: 10.1016/j.nicl.2021.102722 (PMC8209846; doi:10.1016/j.nicl.2021.102722)
Supplement: Supplementary data 1 [file mmc1.docx]

**
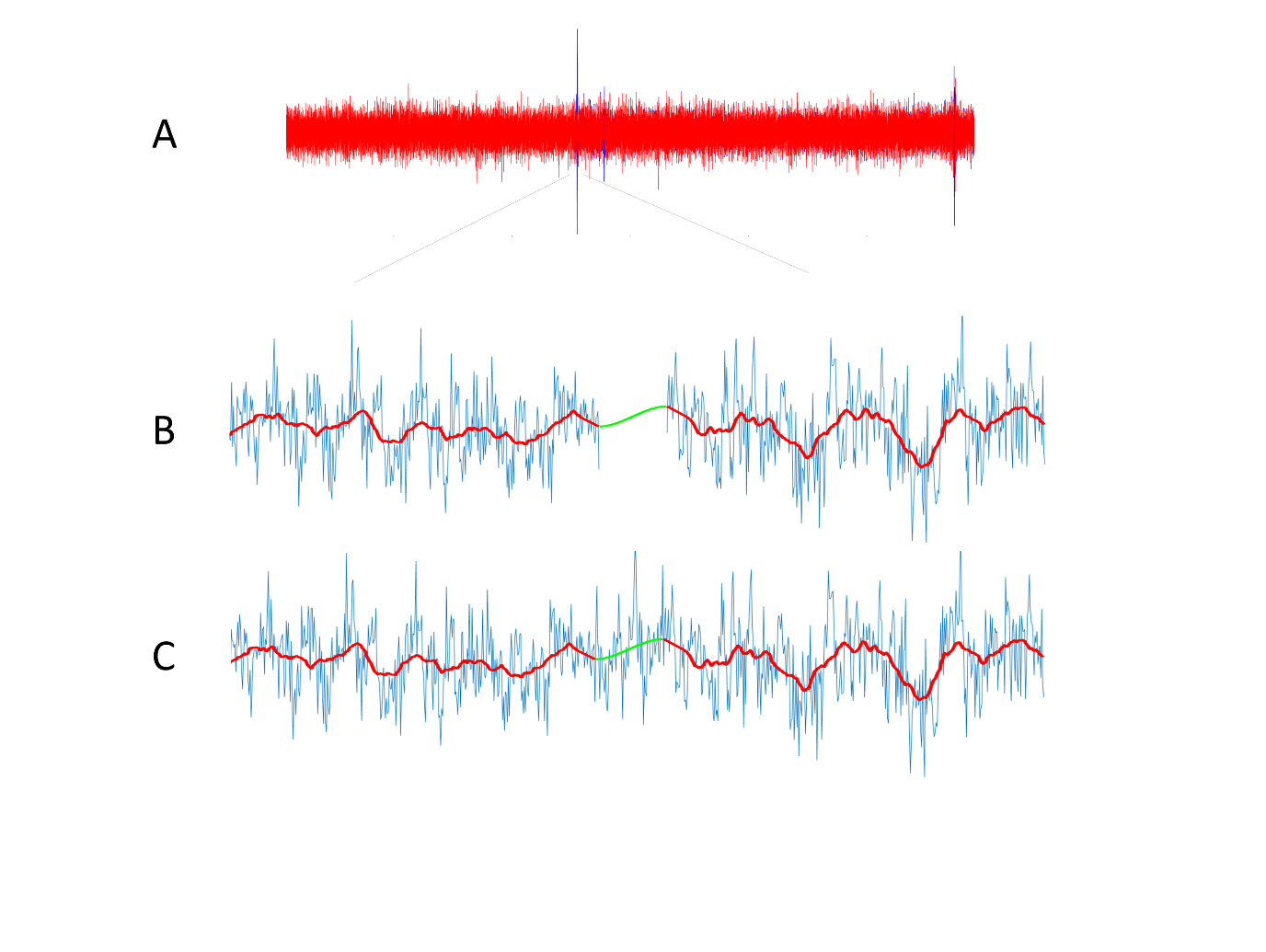
**

**Figure S1. Data replacement process.** (A) Sample points containing high-amplitude artifacts were identified and stored. (B) In a following step, the sample information of the artifacts was used to read-in the continuous raw data excluding the artifactual parts (to avoid additional long-lasting artifacts). (B) The trend of the signal (red line) was obtained with a Savitzky-Golay finite impulse response (FIR) smoothing filter (filter order:1, frame length: 41) and the missing segment was completed with the interp1() MATLAB function, using the shape-preserving piecewise cubic interpolation method (green line). (C) The interpolated values were used to re-trend and insert a clean portion of the data.


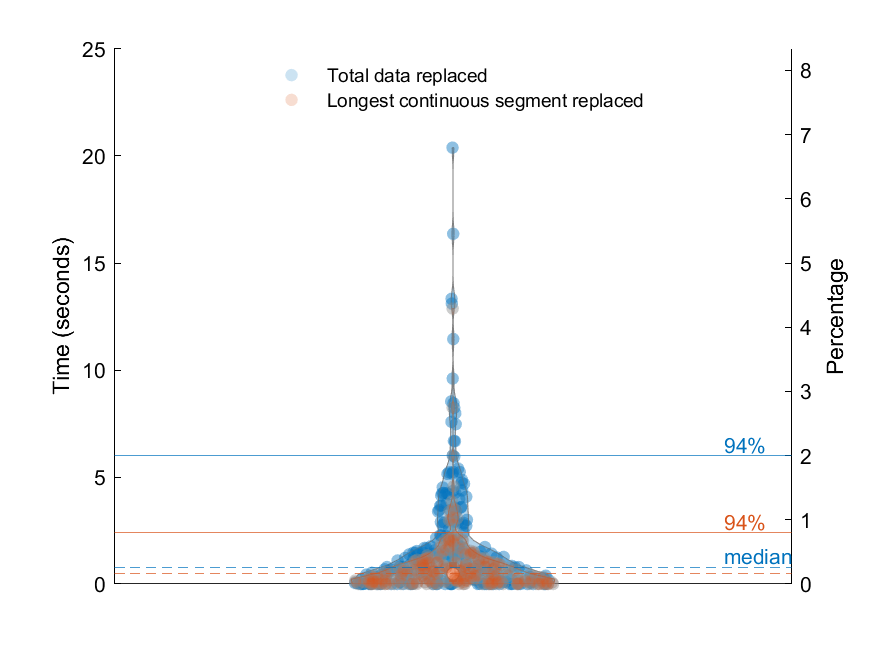


**Figure S2. Summary of the length of the total (blue) and continuous (red) length of data segments replaced.** To maintain the temporal structure of the time series, segments contaminated with high-amplitude noise were replaced with clean segments of the same time series. The median of the longest continuous segments removed was 0.5 seconds (red segmented line) and the median of the total length of data replaced by subject was 0.8 seconds (blue segmented line).


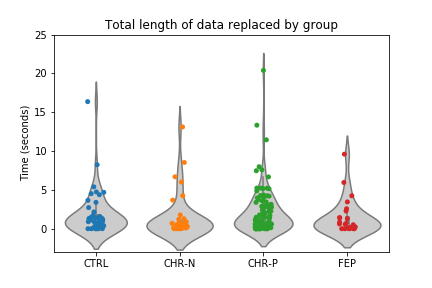


**Figure S3.** Each dot represented the total length of data replaced for each participant, colored by group. The same amount of data was replaced across groups (Kruskal-Wallis independent-sample test, H=6.16, P=0.1)


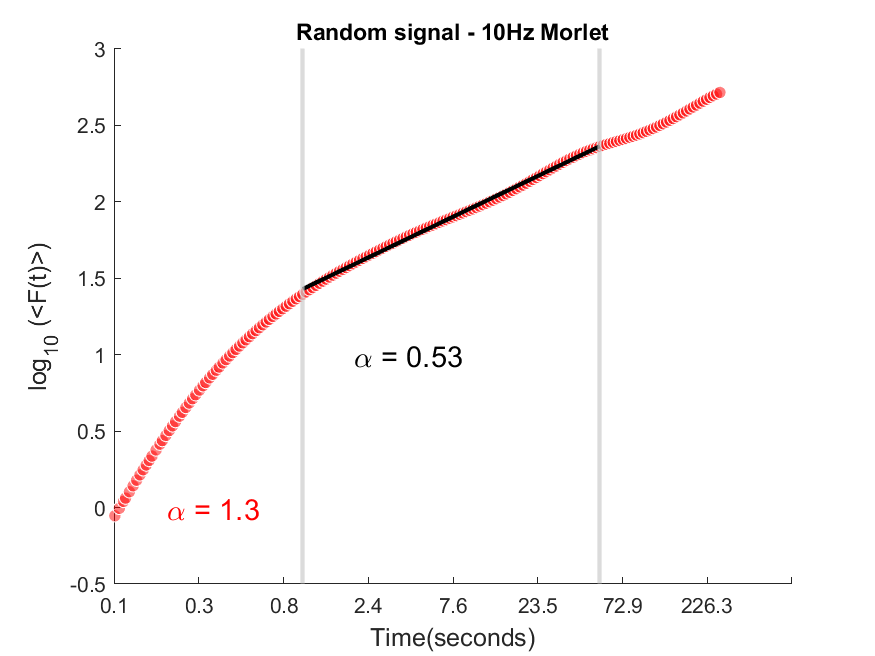


**Figure S4.** A random signal was generated in MATLAB with the same length of the time series analysed in this study (5 minutes). Autocorrelations after 1 second were not affected by the filter, meaning that, fitting of the power law gave a DFA scaling exponent alpha close to 0.5. Time windows shorter than 1 second were notoriously affected by the filter, with a DFA scaling exponent α above 1. To avoid strong autocorrelations induced by the filter, the slope was calculated using window sizes between 1 and 60 second (fitting range indicated with vertical grey lines)


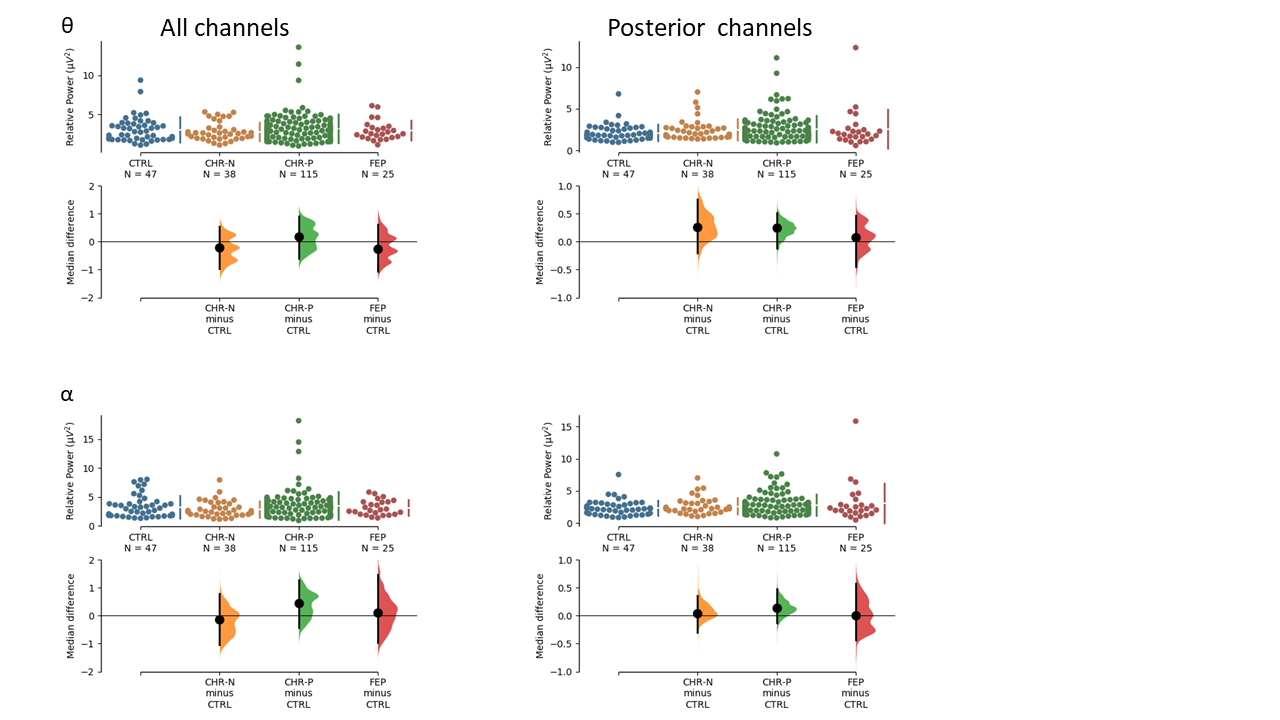


**Figure S5**. Relative power of the clinical groups do not differ from controls in either theta (top row) or alpha (bottom row) frequency bands. Results are similar when either all channels are considered (left column) or only a posterior channel selection (right column).
